# Draft metagenome-assembled genome sequence of a *Dehalogenimonas* species from an enriched consortium for complete trichloroethylene reductive dechlorination

**DOI:** 10.1128/mra.01316-25

**Published:** 2026-03-12

**Authors:** Lei Zhang, Kaiwen Yang, Xiaojun Zhang

**Affiliations:** 1 State Key Laboratory of Microbial Metabolism, School of Life Sciences and Biotechnology, Shanghai Jiao Tong University, Shanghai, China; 2 National Center for Translational Medicine (Shanghai), Shanghai Jiao Tong University 12474, Shanghai, China; Indiana University Bloomington, Bloomington, Indiana, USA

**Keywords:** dechlorination, consortium, metagenome, genome assembly

## Abstract

A draft metagenome-assembled genome was recovered from an anaerobic consortium capable of complete reductive dechlorination of trichloroethene to ethene. The draft genome, assigned to a *Dehalogenimonas* species, is 1.84 Mb in size with a G + C content of 54.53% and encodes 33 reductive dehalogenase homologs.

## ANNOUNCEMENT

Organohalide-respiring bacteria conserve energy via reductive dehalogenases (RDase)-mediated respiratory reductive dehalogenation (1). However, only *Dehalococcoides* and *Dehalogenimonas* are known to completely dechlorinate trichloroethene (TCE) to ethene (1). Although *Dehalococcoides* genomes have been elucidated, genomic information on *Dehalogenimonas* remains limited. A *Dehalogenimonas* metagenome-assembled genome (MAG) was recovered from an anaerobic complete TCE dechlorination enrichment via dilution-to-extinction (2). The original enrichment was established from chloroethene-contaminated soil as previously described (3). The culture was serially diluted in DCB-1 mineral salt medium (2) amended with 20 mM acetate, 1.22 mM hydrogen, and 500 µM TCE, and incubated at 30°C.

DNA was extracted using the DNeasy PowerSoil Pro Kit (QIAGEN, Cat. No. 47014). DNA quality was checked by Quantus fluorometer (PicoGreen). DNA was sheared to approximately 350 bp using a Covaris M220 system (Gene Company Limited, China). Paired-end libraries (NEXTFLEX Rapid DNA-Seq Kit) were sequenced on an Illumina NovaSeq X Plus platform (Illumina Inc., San Diego, CA, USA) using a NovaSeq X Series 25B Reagent Kit. The run generated 74.8 million paired-end reads (11.29 Gb). The raw reads were analyzed with anvi’o-8 platform (4). Reads quality control and filtering were performed using illumina-utils v2.13 (5). Unfiltered raw reads were deposited in the SRA; quality-filtered reads were used for downstream analyses. Taxonomic profiling used KrakenUniq v1.0.4 (6). Reads were assembled with MEGAHIT v1.2.9 (7) using default parameters, and contigs ≥1,000 bp were retained. Reads were mapped with Bowtie2 v2.5.4 (8). Gene prediction used Prodigal v2.6.3 (9). Functional annotation was based on hidden Markov models (HMMs) against KEGG (10), KOfam (11), and Pfam (12) in anvi’o. Putative reductive dehalogenases were identified using the TIGR02486 HMM model. Binning used CONCOCT (13), MaxBin2 (14), and MetaBAT2 (15) with DASTool (16) refinement.

**Fig 1:**
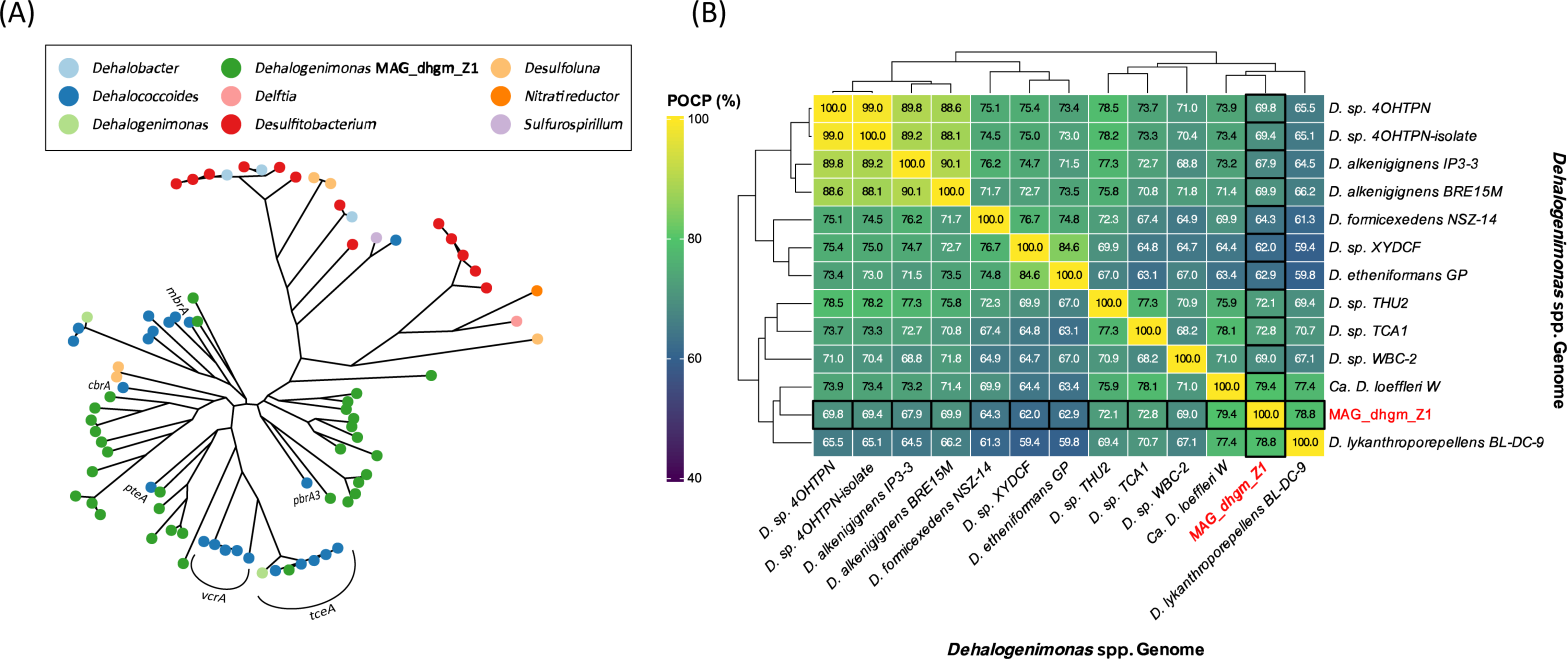
RDase phylogeny and Percentage of Conserved Proteins (POCP) relatedness of MAG_dhgm_Z1. (**A**) Maximum-likelihood phylogenetic tree of 33 putative RDases from MAG_dhgm_Z1 and reference RDases (18, 19) inferred with FastTree (v2.2.0) (20). (**B**) POCP comparisons among related genomes.

*Dehalogenimonas* MAG_dhgm_Z1 genome statistics are summarized in Table 1. Two contigs (totaling ~97 kb) identified as *Proteobacteria*-derived contaminants by NCBI’s contamination screening were removed prior to final submission. A total of 1,858 coding sequences were predicted, including 33 putative RDase genes. These RDases were protein aligned with MAFFT (v7.526) (17) to reference RDases (18, 19) (Fig. 1A).

**TABLE 1:** Basic genomic features of *Dehalogenimonas* MAG_dhgm_Z1

No. of contigs	*N*_50_	GC content	Total size	Completeness	Redundancy
61	48,002	54.53%	1.84 Mb	97.18%	1.41%

HMM analysis identified one 16S rRNA gene and one 23S rRNA gene, and the 16S rRNA gene showed 99.46% identity with *Dehalogenimonas lykanthroporepellens* strain BL-DC-9ᵀ (CP002084.1). Average nucleotide identity (ANI) and Percentage of Conserved Proteins (POCP) were calculated between the MAG and all available *Dehalogenimonas* complete genomes from NCBI using fastANI (21) v1.34 and POCP-nf pipeline (22, 23), respectively. The highest identity was 83.66% with *D. lykanthroporepellens* BL-DC-9ᵀ, with lower similarities to other related strains, including Candidatus *D. loeffleri* strain W (78.04%), strain IP3-3ᵀ (75.98%), and strain NSZ-14ᵀ (75.6%). The POCP values supported assignment to the genus *Dehalogenimonas* (Fig. 1B). These values indicate that *Dehalogenimonas* MAG_dhgm_Z1 constitutes a distinct lineage within the genus *Dehalogenimonas*, expanding the *Dehalogenimonas* pangenome.

## Data Availability

The draft metagenome-assembled genome (MAG) sequence of Dehalogenimonas MAG_dhgm_Z1 has been deposited in European Nucleotide Archive (ENA) under accession GCA_978046095.1. The associated ENA Study and Sample accessions are PRJEB104910 and SAMEA120968246, respectively. The raw sequencing reads are available under ENA Run, accessions ERR16125411 and ERR16125412.
